# The ICOSL Expression Predicts Better Prognosis for Nasopharyngeal Carcinoma via Enhancing Oncoimmunity

**DOI:** 10.1155/2020/9756732

**Published:** 2020-01-07

**Authors:** Geng Zhang, Yi Xu, Sen Zhang, Huifang Zhou

**Affiliations:** ^1^Department of Otorhinolaryngology, Tianjin Medical University General Hospital, Tianjin 300052, China; ^2^State Key Laboratory of Bioactive Substances and Functions of Natural Medicines, Institute of Materia Medica, Chinese Academy of Medical Sciences & Peking Union Medical College, Beijing 100050, China

## Abstract

Nasopharyngeal carcinoma (NPC) is a malignant tumor with poor prognosis, high morbidity, and mortality. Currently, immunocheckpoint therapy has led to new treatment strategies for almost all cancers, including nasopharyngeal carcinoma. Inducible T-cell aggregation ligand (ICOSL) belongs to the b7-cd28 immunoglobulin superfamily, which is a ligand of ICOS, and also begins to draw attention for its potential usage in cancer treatment. Previous studies from our laboratory have suggested that ICOS expression in tumor-infiltrating lymphocytes is correlated with beneficial outcome, but little is known about the role of ICOSL in NPC. In the current study, ICOSL expression in NPC tumor sections was stained by immunohistochemistry (IHC), and both lymphatic metastasis and distant metastasis showed decreased expression, which was negatively correlated with TNM stage of nasopharyngeal carcinoma. Importantly, high ICOSL expression was significantly associated with overall survival (OS) in patients with NPC (*n* = 225, *p* < 0.001), and multivariate analysis confirmed that high ICOSL expression was an independent prognostic factor. Fresh nasopharyngeal carcinoma specimens were excised, and the specific expression of cytokines was analyzed by enzyme-linked immunosorbent assay (ELISA). The expression level of ICOSL is positively correlated with interferon-gamma (IFN-*γ*) concentration in tumor tissues, which is characteristic of T helper 1 (Th1) cells. Knocking down ICOSL by RNAi did not influence the proliferation, migration, and invasion ability of NPC cells. Conclusively, ICOSL expression is associated with increased survival rate in patients with nasopharyngeal carcinoma, which may be a clinical biomarker for prognosis of nasopharyngeal carcinoma.

## 1. Introduction

Nasopharyngeal carcinoma (NPC) is a local malignant tumor, mainly distributed in southern China, with an annual incidence of nearly 30/100,000 [1]. More than 70 percent of newly diagnosed nasopharyngeal carcinoma patients are locally advanced [2]. With the application of concurrent chemoradiotherapy, intensity-modulated radiotherapy (IMRT), and imaging techniques, local area control has been greatly improved, and distant metastasis has become the main cause of treatment failure in nasopharyngeal carcinoma [3]. Although there are multiple biomarkers for evaluating the prognosis of recurrent nasopharyngeal carcinoma, the overall survival rate (OS) has not improved, and the 5-year OS is only 30% [4, 5], making the treatment of recurrent nasopharyngeal carcinoma being a major clinical challenge [6, 7]. Therefore, there is an urgent need to find reliable prognostic markers and effective treatments.

Immunocheckpoint immunotherapy has brought a deep revolution for cancer treatment; therefore, investigating more immunocheckpoint proteins or pathways is a hot point [8]. ICOSL-ICOS axis is one of the important costimulatory axes in the immune system [9]. Previous studies from our laboratory demonstrate that ICOS + cell infiltration of nasopharyngeal carcinoma is beneficial to improve prognosis [10]. By immunohistochemistry, we demonstrate that ICOS expression is decreased in both lymphatic metastasis and distant metastasis and is negatively correlated with TNM staging of nasopharyngeal carcinoma. Importantly, elevated levels of ICOS expression are significantly associated with overall survival (OS) in patients with nasopharyngeal carcinoma (*p* < 0.001). ICOS expression is also an independent prognostic factor by multivariate analysis. The expression level of ICOS is related to the increase of the number of cytotoxic T lymphocytes, the expression of high IFN-*γ*, and the characteristics of Th1 cells [10]. However, about the sole ligand of ICOS, there is still lack of in-depth studies about its role in the NPC.

The interaction of ICOS with its unique ligand and the induction of T-cell costimulatory ligands (ICOSL; also known as b7-associated protein-1) trigger key activities of T cells, including cytokine production and differentiation into T-follicular helper cells (Tfh) cell line effector T cells [11]. It was originally thought to be limited to the T-cell activation phase and T-cell-dependent B-cell response [12]; however, in recent studies, the interaction between ICOS and ICOSL has been shown to play a role in the downstream survival and expansion of T cells (effectors and modulators) and thus could influence the immune-mediated cancer tolerance [13, 14]. Importantly, the growing understanding of ICOSL biology has now been translated into its use as a viable therapeutic target. Successful completion of phase I clinical studies of patients with systemic lupus erythematosus and phase II clinical trials of patients with Sjogren's syndrome (both performed by Amgen Inc.) demonstrated the effectiveness of human anti-ICOSL mAb prezalumab [15, 16]. The importance of ICOSL and its role in antibody-mediated diseases has also been confirmed in preclinical models of several human diseases including RA, SLE, and uveitis [17, 18]. This pathway promotes cytokines such as T cells, IL-10, IL-4, IL-5, IFN-*γ*, and IL-17, depending on the dominant role of cell type [19].

In the previous study, the expression pattern of ICOS in the NPC has been well investigated by our laboratory [10], and we demonstrated that higher expression of ICOS in NPC tumor tissues is associated with improved survival of NPC patients. Percentage of ICOS^+^ cells acting as Th1 cells in primary tumor tissue may be a clinical biomarker for good prognosis of NPC patients [10]. This study used the same batch of nasopharyngeal carcinoma tissue sections to study the expression of ICOSL in nasopharyngeal carcinoma and its clinical significance. In addition, ICOSL was knocked down in NPC cells by RNA interference (RNAi), and to investigate the role of ICOSL in proliferation, migration, and invasion ability of NPC cells.

## 2. Materials and Methods

### 2.1. Patients and Samples

The study was approved by the Independent Ethics Committee of Tianjin Medical University General Hospital. The patient's medical record is identified and anonymized prior to analysis.

In this study, from March 2012 to May 2015, 270 patients with nasopharyngeal carcinoma were consecutively enrolled in our hospital, the same as previously published [10]. Patients were selected according to the following criteria: (1) histologically confirmed locally advanced nasopharyngeal carcinoma and biopsy specimens available; (2) Karnofsky score ≥70 points; (3) radical IMRT at the time of initial diagnosis and concurrent cisplatin chemotherapy; (4) no malignancy tumors or other concomitant malignant diseases; and (5) ICOSL staining can be detected in tumor tissues. Therefore, there were 225 patients who met the conditions of this study. All patients underwent disease staging using the American Joint Committee on Cancer (AJCC) 2010 staging system. The clinical features are shown in Table 1. All patients were enrolled in the same treatment strategy, IMRT combined with cisplatin chemotherapy. A biopsy specimen was taken by nasal endoscopy for pathological analysis.

### 2.2. Immunohistochemistry (IHC) Staining

Immunohistochemical staining of 5 *μ*m partial formalin-fixed paraffin-embedded nasopharyngeal biopsy specimens was carried out in our hospital's pathology department with antibodies 1 : 200 anti-ICOSL (Abcam, Danvers, MA, USA) using standard protocols for routine diagnostic specimens. Hematoxylin and eosin sections were also used to examine the presence of tumors. The expression of ICOSL was evaluated based on the degree and intensity of staining. Positive area was measured using Image-Pro software and expressed as a percentage of each HPF-positive area. Extent was classified as 0, ≤5%; 1, 6 to ≤15%; 2, 16 to ≤30%; 3, 31 to ≤50%; 4, 50%–75%; and 5, >75%. Intensity of staining was classified as 0, negative; 1, weak; 2, moderate; and 3, strong based on professional experience by two pathologists. The cutoff value of the low expression group and the high expression group was determined by the degree × intensity staining level and the final score. Then, the protein expression is divided into four categories: 0–3 is represented as “-”, 4–6 as “+”, 7–9 as “++”, >and 9 as “+++”;The final score <6 is defined as low expression, and the final score ≥6 is defined as high expression.

### 2.3. Isolation and Culture of CD4^+^ and CD8^+^ T Cells

Purified CD4^+^ T cells, isolated from PBMNCs of healthy donors using MACS CD4^+^ T-cell isolation kit (Miltenyi Biotec, Bergisch Gladbach, Germany), were seeded at 5 × 104 cells/well on 96-well plates coated with 1 *μ*g/ml anti-CD3 monoclonal antibody (eBiosciences, San Diego, CA, USA) and stimulated with 3 *μ*g/ml anti-CD28 monoclonal antibody (eBiosciences) and 20 ng/ml IL-2 for 5 days. Full-length hICOSL cDNA was cloned into eukaryotic expression vector GV230. HEL or HL-60 cells were transfected transiently with the positive clones of constructed recombinant plasmid GV230-hICOSL and were subsequently screened with 800 *μ*g/ml G418 for 7 days. qPCR and western blot were performed to determine the mRNA and protein levels of ICOSL to confirm the overexpression of ICOSL. After treatment with 20 *μ*g/ml mitomycin for 1 h, AML cells or AML overexpressed ICOSL was cocultured with CD4^+^ T cells at a ratio of 1 : 1 for 5 days in the presence or absence of 10 *μ*g/mL neutralizing anti-ICOSL antibody (eBiosciences), with stimulation with plate-coated anti-CD3 (1 *μ*g/ml) and soluble anti-CD28 (3 *μ*g/ml) and IL-2 (20 ng/ml).

### 2.4. ELISA for Analyzing the Cytokines in the Tumor Tissues

Previous studies have shown that ICOSL-ICOS signaling plays a role mainly by activating effector T cells to regulate cytokine production [20], and it induces T cells to produce cytokines such as IFN-*γ* (Th1 marker), IL-4 (Th2 marker), IL-17 (Th17 marker), and IL-10 (Treg marker). Therefore, ELISA was used to test the concentration of cytokines in the tumor tissues, and procedure was followed using the previously described procedure [21]. Briefly, these four cytokine ELISA kits were purchased from Abcam Inc. (California, USA), fresh tissue was homogenized with 1 : 20 (by weight: volume) PBS, and PBS homogenate was added to 96-well plates according to the commercial menu. Cytokine concentrations were obtained by means of a standard curve. All cytokine concentrations were calculated per 100 *μ*g of total protein.

### 2.5. Cell Culture and Cell Proliferation, Migration, and Invasion Assay by Knocking Down ICOSL in NPC Cells

Human NPC cell lines C666, HNE-3, CG1, and C666-1 were purchased from the Cell Center of Chinese Academy of Medical Sciences (Beijing, China) and cultured in RPMI Media 1640 (Thermo Fisher Scientific, Inc., Waltham, MA, USA) supplemented with 10% fetal bovine serum (Sigma-Aldrich, St. Louis, MO, USA). Cells were cultured in a 37°C incubator at 5% CO_2_.

Targeting ICOSL interference RNA was synthesized by Life Technologies (Carlsbad, CA, USA) and transiently transferred into NPC cell lines, but the interference sequence is not available in Life Technologies. After that, proliferation assay, wound-healing assay, and transwell assay were conducted to investigate the influence of ICOSL on NPC cells.

Cell proliferation ability was measured by MTT assay. Logarithmic growth phase cells were taken, digested with 0.25% trypsin, and diluted to 5 × 10^4^ cells/ml suspension in 10% fetal bovine serum medium. Cells were seeded in 96-well plates with 100 *μ*L each. Five parallel wells were prepared and cultured as a blank control. After being cultured for 72 hours, the supernatant was discarded and supplemented with 100 *μ*L of MTT for 4 hours. DMSO (Sigma-Aldrich) was added, and the OD value at 490 nm was measured by Labsystems WELLSCAN MK3 ELISA (Dragon, Finland).

For the wound-healing assay, 6-well plate was used. Briefly, 5 horizontal lines were drawn evenly on the back of the plate. 5 × 10^5^ cells (200 *μ*l) were added in each well and the medium was covered evenly. After being cultured for 24 hours, a vertical line was drawn by the pipette tip (200 *μ*L) on the plate. Suspended cell was washed by PBS 3 times. Cells were cultured in incubator. Image was captured 0, 12 and 24 hours after culture.

Cell invasion assays were performed using Corning™ Biocoat™ Matrix™ Invasion Chamber and Corning™ Matrigel Matrix (Thermo Fisher Scientific, Waltham, MA, USA). A concentration of 100 *μ*L of 5 × 10^5^/ml was added to the upper chamber (8 *μ*m pores), and 600 *μ*L of medium containing 10% serum was added to the house. After 6 hours of culture, the culture solution was taken out, and the nonmigrated cells were swabbed. The cells were fixed in 4% polyoxymethylene for 10 minutes and stained with crystal violet for 10 minutes. The filter was sealed with a neutral gel and photographed under an inverted microscope (200x). Cell counts were performed using Image-Pro + version 6 software, with 3 wells per group, and 5 fields of view per well were randomly selected to calculate the average number of cells.

### 2.6. qRT-PCR

qRT-PCR was used to evaluate the knocking down efficiency. Briefly, total RNA was extracted from kidney tissue using TRIzol reagent (Life Technologies, Inc., USA) according to the manufacturer's instructions; RNA (10 *μ*g) from each group was reverse-transcribed by reverse-transcribed kit (ReverTra Ace R qPCR RT Toolkit, Toyobo Inc., Japan) to get the corresponding complement. The Thunderbird qPCR RT-qPCR was used for mixing (Toyobo Inc., Japan) in the 7900 ABI Prism Sequence Detection System (Applied Biosystems, Inc., Calcium, USA), and ΔCt normalized to the house keeping gene, GAPDH. The expression is calculated as a fold change of 2^ΔCt(treated−untreated)^. The forward primer sequence of ICOSL was 5′ GTTTCACTGCCTGGTGTTGAGC3′, and the reverse primer sequence of ICOSL was 5′ ACGACGGGCACGCTGAAGTTTG3′. The forward primer sequence of actin was 5′CCTGGACTTCGAGCAAGAGAT3′ and its reverse primer sequence was 5′GCCGATCCACACGGAGTACT3′. The qPCR cycle conditions were as follows: one cycle lasted for 30 seconds at 95°C, 40 cycles lasted for 15 seconds at 95°C, 60°C for 30 seconds, and 72°C for 30 seconds.

### 2.7. Western Blot

Lysed NPC cells were used for protein extraction. Protein was extracted with RIPA buffer containing 1 mM PMSF (Sigma, USA). Concentrations were determined using a bicinchoninic acid assay kit (Pierce, Rockford, IL). The same amount of protein (10 *μ*g/lane) was subjected to SDS-polyacrylamide gel electrophoresis (SDS-PAGE) at a constant voltage of 80 V for 120 minutes. The protein was transferred to a polyvinylidene fluoride (PVDF) membrane (Millipore, Bedford, MA, USA). The membrane was blocked with 5% skim milk in Tris-buffered saline (TBS) for 1 hour at room temperature. After blocking, the membrane was incubated with primary antibody, including anti-ICOSL (Abcam, Danvers, MA, USA) and anti-beta-actin antibody (Abcam) overnight at 4°C. The next morning, the membrane was washed with TBS containing 0.05% Tween-20 (TBST) and probed with horseradish peroxidase-conjugated secondary antibody for 1 hour at 37°C. After washing with TBST, the membrane was developed with an enhanced chemiluminescence (ECL) kit and visualized by a LAS 4000 imaging system (GE Healthcare, USA). Bands were quantified by ImageJ software by measuring the intensity of target gene bands normalized to internal control *β*-actin. All reactions were performed in duplicate, and experiments were repeated to ensure repeatable results.

### 2.8. Statistical Analysis

Statistical analysis was performed using the Social Science Statistics Package 25.0 (SPSS, Chicago, IL, USA) software. Disease-free survival (DFS) and overall survival (OS) were estimated using the Kaplan–Meier method. DFS and OS were measured from the first day of treatment to the day of the event. Univariate analysis was performed using a log-rank test. The exact test of *χ*^2^ and Fisher was used to compare the difference between the ICOSL high group and the ICOSL low group. Multivariate analysis was performed using the Cox proportional hazard model. All statistical tests were bilateral tests; *p* < 0.05 for the difference was considered as statistically significant.

## 3. Results

### 3.1. General Information

Of the 225 patients enrolled, 149 were males and 76 were females with an average age of 50 years (range, 20–74 years). The median body mass index (BMI) was 22.4 kg/m^2^ (range 16.0–32.5 kg/m^2^). A total of 139 patients were infected with EB virus, and the infection rate was 61.7%. All tumors were nonkeratinized phenotypes. After a median follow-up of 70 months, 21 (9.3%) patients died, and 15 (6.7%), 11 (4.9%), and 13 (5.8%) patients experienced local failure, lymph node metastasis, and distant metastases. The 5-year DFS and OS were 55.8% and 59.3%, respectively. Patient detailed characteristics are shown in Table 1.

### 3.2. Clinicopathologic Correlations with ICOSL

**Figure 1:**
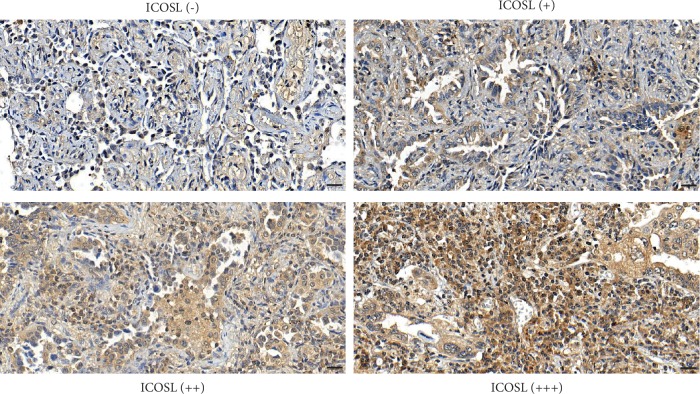
Immunohistochemical staining for ICOSL in patients with NPC, and classification of different intensity of ICOSL staining.

ICOSL staining was detectable in specimens from all patients and was mainly located in the cytoplasm and extracellular matrix in the tumor tissues. ICOSL staining is divided into high, medium, and low expression and no staining. Representative ICOSL staining in nasopharyngeal carcinoma is shown in Figure 1. In this study, high expression of ICOSL staining was not significantly associated with clinical-pathological parameters such as age, gender, BMI, and EBV infection, but was significantly associated with clinical diagnosis, lymphocyte metastasis, and distant metastasis. Specific data are shown in Table 1.

### 3.3. Prognostic Value Associated with ICOSL

**Figure 2:**
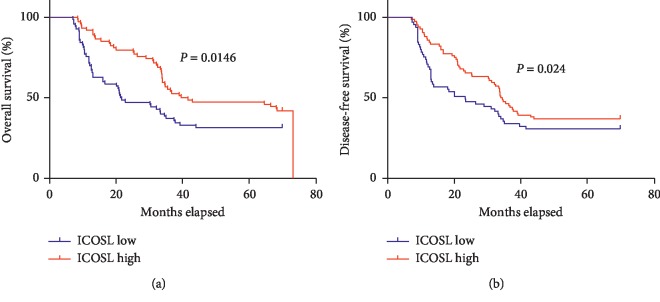
Kaplan–Meier curves stratified by the patterns of immunohistochemical staining for ICOSL (low vs. high).

The predictive value of various potential prognostic factors such as age, gender, BMI, total stage, and ICOSL for OS and DFS was assessed. The results of single-factor and multivariate analysis are shown in Table 2. Our results indicate that low expression of ICOSL is associated with worsening of OS and DFS (5y OS: 49.4% vs. 65.7%, *p*=0.0146; 5y DFS: 45.9% vs. 62.4%, *p*=0.024, Figure 2) In the univariate and multivariate analyses, ICOS was considered to be an independent prognostic factor for OS (one-way hazard ratio 2.865, multifactor hazard ratio 2.475, both *p* < 0.01) (see Table 2 for specific data).

### 3.4. ELISA Showed That the Expression of ICOSL Was Positively Correlated with IFN-*γ* Level in NPC Tumors

**Figure 3:**
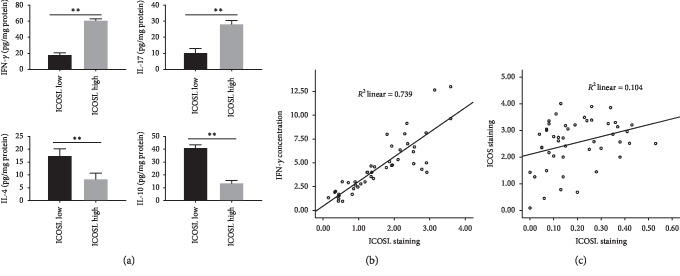
Four kinds of cytokine concentration from tumor tissues in ICOSL higher or lower groups by ELISA analysis, and ICOSL expression was positively correlated with IFN-*γ* concentration in tumor tissues and ICOS + infiltration cells.

As shown in Figure 3(a), in the ICOSL high expression group, the concentration of IFN-*γ* and IL-17 from tumor tissues was significantly higher than that in the low expression group of ICOSL. In contrast, IL-4 and IL-10 were significantly lower in the ICOSL high expression group than in the low expression group. Regression analysis showed a positive correlation between IFN-*γ* and ICOSL expression (Figure 3(b)). Also, ICOSL expression was correlated with ICOS^+^ cell infiltration (Figure 3(c)).

### 3.5. Knocking Down ICOSL Did Not Change the Biofunction of NPC Cells

**Figure 4:**
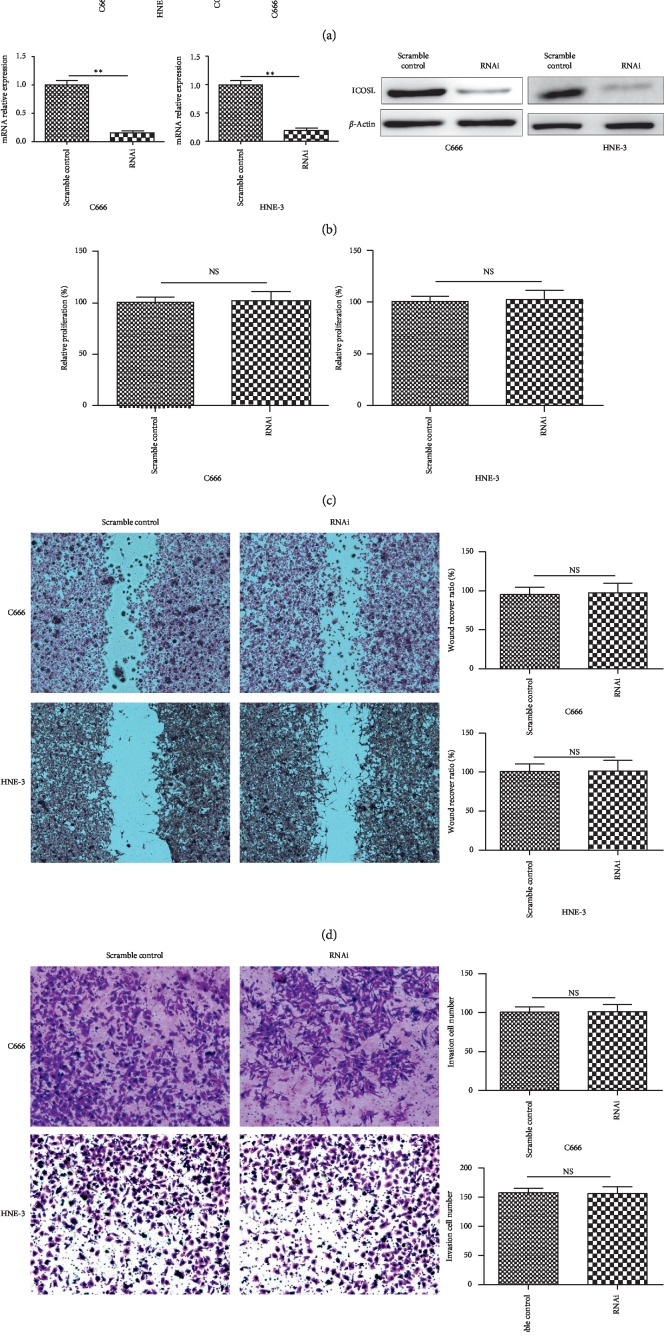
Knocking down ICOSL did not change the proliferation, migration, and invasion ability of NPC cells in vitro. (a) mRNA and protein levels from four NPC cell lines; (b) qRT-PCR and western blot prove that ICOSL siRNA strongly reduces the ICOSL production in NPC cell in both mRNA and protein levels; (c) MTT assay shows that reduced ICOSL does not change the proliferation ability of NPC cells; (d) reduced ICOSL did influence the migration capability of NPC cells; (e) reduced ICOSL did influence the invasion capability of NPC cells.

Both real-time PCR and western blot demonstrate that C666 and HNE-3 have higher ICOSL expression compared with CG1 and C666-1 (Figure 4(a)); therefore, C666 and HNE-3 had been selected for RNA knocking down experiment. By siRNA transfection, reduction of ICOSL mRNA expression was confirmed by RT-PCR and ICOSL protein level was confirmed by western blot (Figure 4(b)). By MTT assay, wound-healing assay, and transwell assay, reducing ICOSL expression did show any significant difference between siRNA cells and scramble control cells (Figure 4(c)–4(e)).

## 4. Discussion

In our study, different ICOSL expression patterns were noticed in tumor sections among different stages of NPC patients. Like ICOS expression, higher expression of ICOSL predicts beneficial prognosis for NPC patients [10], which is a continuity from the previous study of ICOS expression pattern in NPC from our laboratory. To our knowledge, our study describes for the first time ICOSL expression in NPC patients due to rare occurrence of NPC in Western countries. Using the same batch of NPC patients, by regression analysis, we confirm that ICOSL expression is significantly correlated with ICOS + T lymphocyte infiltration in NPC tumor tissues, which suggests that ICOSL in NPC tissues might activate ICOS expression and enhance ICOS + cell infiltration.

Our results suggest that higher expression of ICOSL in NPC is significantly associated with improved overall survival and disease-free survival. Based on known immunological studies, ICOSL interacts with ICOS which was distributed in the cell membranes of T cells and consequently activates the production either T effector cells or Treg cells [22, 23]. Which one is dominated, effector T cell or Treg cells, depends on tumor type or tumor microenvironment. As shown in Figure 3, by ELISA test, we confirm that ICOSL-ICOS activation promotes T effector production, including Th1 and Th17 polarization, not IL-4 and IL-10. As we can see in the NPC tissues, IFN-*γ* and IL-17 increased in ICOSL higher group, and on the contrary, IL-4 and IL-10 decreased in ICOSL higher group. We hypothesize that ICOSL-ICOS axis selectively activates effector T cells in NPC tumors, especially cytotoxic T cells. That partially explains why ICOSL can predict longer overall survival and DFS in NPC.

The role of ICOSL/ICOS axis in cancer development and progression seems to be distinguished in different cancers. Previous research reports that melanoma cells express ICOS ligand to promote the activation and expansion of T regulatory cells and enhance the IL-10 production of Treg cells [24]. It is one of the mechanisms of immune evasion for melanoma [24]. Same immunosuppressive effect of ICOSL has also been reported in breast cancer, in which ICOSL/ICOS expression was positively correlated with Treg infiltration [25]. However, there are also several controversy reports about ICOSL role in cancer. Jeffrey also reported that ICOSL enhances antitumor responses of CD8^+^ T cells in vitro by using fibrosarcoma cell line Sa1N with exogenous ICOSL expression [26]. Liu et al. demonstrated that ICOSL enhances tumor susceptibility to T-cell therapy and may be able to tap into the potential of the large number of tumor-specific T cells in the melanoma patients by activating B7H-mediated signal transduction within the tumor milieu [27].

Most NPC cells express ICOSL cells and, not like ICOS, only exist in the cell membranes of portion of infiltrated T cells. To understand the role of ICOSL on NPC cells, the ICOSL was knocked down in two NPC cells which had higher baseline ICOSL expression. By knocking down ICOSL from C666 and HNE-3, we did not find significant difference between RNAi group and scramble group on proliferation and migration ability. These results suggest that ICOSL itself did not influence the physiology of NPC cells. The reason why higher expression of ICOSL could predict better prognosis is that ICOSL possibly activates ICOS and then initiates production of cytotoxic T cells and effector T cells. This mechanism is similar to current popular oncoimmunotherapy. Several previous studies have shown that ICOS^+^ cells as Th1 cells are associated with improved survival in patients with colorectal cancer [28]. Compared with other costimulatory receptors of CD28 in this family, ICOS has little effect on naive T cell activation and T-cell proliferation [17, 29, 30]. ICOS signaling plays an important role in activation and effector T-cell regulation of cytokine production, especially Th1 and Th2 effect responses [22]. Since Th1 and Th2 cells play almost the opposite role in the immune system, disruption of ICOS may lead to different outcomes, depending on the effector function that ICOS plays a leading role. Our data suggest that higher expressed ICOSL tumor tissues express higher levels of IFN-*γ* and IL-17, and less IL-10 and IL-4, compared with ICOSL low expressed tumor tissue, indicating that ICOSL promotes Th1 and Th17 response in NPC patients.

These results are consistent with the previous research. Previous studies have reported that Th1 cells inhibit tumor cell invasion and metastasis by communicating with tumor-associated myeloid cells, including TAMs and MDSCs [31], which may help to improve the survival of patients with nasopharyngeal carcinoma with high ICOSL expression. The mechanism by which ICOSL signaling regulates Th1 effector response remains to be further studied.

Currently, ICOSL has become a drug target for tumor immunotherapy and autoimmune diseases. By the current study, inhibiting the ICOSL seemingly did not change the biofunction of NPC cell itself, suggesting ICOSL influences oncogenesis and tumor progression possible only by enhancing the cytotoxic immunity. However, ICOSL immunohistochemistry is easier to be observed; therefore, ICOSL staining is possibly useful than ICOS staining in the clinical practice in the future. However, lack of direct evidence of influence of tumor cell-associated ICOSL on CD4^+^ and CD8^+^ T cells is a limitation for this current study, and more detailed molecular study is needed in the further study.

## 5. Conclusion

The expression level of ICOSL in nasopharyngeal carcinoma is positively correlated with OS, which may be due to its costimulatory effect enhancing the cytotoxicity of Th1 and Th17. The role of ICOSL in the NPC deserves further study in the future.

## Figures and Tables

**Table 1:** Patient characteristics and significance of ICOSL expression in clinical parameters.

Characteristic	High expression (*n* = 136)	Low expression (*n* = 89)	*p* value
Sex			0.888
Male	91	68	
Female	45	31	
Age			0.475
≥50	86	61	
<50	50	28	
BMI (kg/m^2^)			0.879
≥23	52	27	
<23	84	41	
WHO histologic type			0.201
Differentiated	44	37	
Undifferentiated	92	52	
EBV infection			0.266
Positive	80	59	
Negative	56	30	
T classification			0.024
T1 + T2	94	48	
T3 + T4	42	41	
Lymph node metastasis			0.020
Absent	82	39	
Present	54	50	
Distant metastasis			0.038
Absent	130	78	
Present	6	11	
Overall stage			*p* < 0.001
I + II	93	40	
III + IV	43	49	

BMI: body mass index; EBV: Epstein–Barr virus.

**Table 2:** Univariate and multivariate analyses of prognostic parameters for survival in 225 NPC patients.

Prognostic parameter	Univariate analysis		Multivariate analysis			
	HR	95% CI	*p* value	HR	95% CI	*p* value
Expression of ICOSL (low vs. high)	2.865	1.284–3.621	0.001	2.475	0.987–3.701	0.001
Age (y)	1.512	0.814–1.722	0.049	—	—	—
Sex (male vs. female)	1.894	0.921–2.422	0.032	—	—	—
Tumor differentiation	1.545	0.713–1.934	0.096	1.857	0.756–1.875	0.077
T classification	2.870	1.274–3.897	0.014	2.947	0.941–2.356	0.022
Lymphatic metastasis (present vs. absent)	4.231	2.287–5.214	0.003	4.985	2.241–5.877	0.001
Distant metastasis (present vs. absent)	5.121	3.038–7.145	0.001	5.636	2.514–7.031	0.001

BMI: body mass index; HR: hazard ratio; CI: confidence interval.

## Data Availability

The data used to support the findings of this study are available from the corresponding author upon request.
